# Challenges of Hip Arthroplasty in a Paretic, Spastic Limb: A Case Study on Managing Femoral Neck Fracture Following Fixation Failure in a Hemiparetic Patient

**DOI:** 10.3390/jcm13144023

**Published:** 2024-07-10

**Authors:** Izabela Dąbkowska, Lena Sobiech, Michał Merkisz, Karolina Turżańska, Tomasz Blicharski, Katarzyna Jankiewicz

**Affiliations:** 1 Department of Sports Medicine, Faculty of Health Science, Medical University of Lublin, Chodźki 15, 20-093 Lublin, Poland; 2 Department of Rehabilitation and Orthopedics, Medical University of Lublin, Jaczewskiego 8, 20-954 Lublin, Polandtomasz.blicharski@umlub.pl (T.B.); 3 2nd Department of Gynaecology, Medical University, Jaczewskiego 8, 20-954 Lublin, Poland; katarzyna.jankiewicz@umlub.pl

**Keywords:** total hip replacement, hemiparesis, interdisciplinary approach, botulinum toxin, rehabilitation, pertrochanteric fracture

## Abstract

**Background:** Hip fractures and strokes are prevalent and escalating issues in geriatric healthcare. The absence of standardized surgical protocols for patients with spastic hemiparesis and multiple comorbidities presents a significant medical challenge. **Methods:** This case study describes a 64-year-old male patient with left-sided hemiparesis and failed surgical treatment of a pertrochanteric fracture in a spastic limb. The patient was admitted to the Department of Rehabilitation and Orthopedics in December 2022 for diagnostics and to establish a treatment plan after five months of non-ambulatory status. **Results:** This study emphasizes the crucial role of preoperative preparation, involving botulinum toxin injections into spastic muscles and physiotherapy, to enhance the supportive function of the paretic limb and improve locomotion before prosthetic surgery. **Conclusions:** The management of hip fractures in patients with spastic paralysis requires a multidisciplinary approach and the development of standardized treatment protocols. This case underscores the importance of comprehensive pre- and postoperative rehabilitation to improve patient outcomes. Further research is needed to establish standardized rehabilitation protocols for spastic patients undergoing hip arthroplasty. Randomized controlled trials could provide valuable insights into the efficacy of various interventions.

## 1. Introduction

Hip fractures and strokes are prevalent and escalating issues in geriatric healthcare. With the global aging population, approximately 1.5 million hip fractures and 14 million strokes occur annually. The projections indicate that these numbers will rise, with proximal femur fractures expected to reach 2.5 million by 2025 and 4.5 million by 2050 [1,2,3,4,5,6,7,8]. Stroke is a significant risk factor that increases the risk of femur fractures in the paretic limb due to sensory disorders, weakened muscle strength, increased muscle tension, impaired balance, and cognitive dysfunction. The literature reports that 7.3–15.3% of stroke patients experience femoral fractures, with their risk of hip fractures being 2–4 times higher than that of healthy individuals [6,9]. Proximal femur fractures significantly impair patients’ quality of life by limiting locomotion and mobility. Prolonged immobility in stroke patients leads to permanent joint stiffness, muscle atrophy, and systemic issues such as cardiorespiratory disorders, significantly increasing their mortality risk [10,11,12]. These patients require continuous care and medical supervision, which impose substantial economic and social burdens, emphasizing the necessity for effective management strategies [5]. In this article, we will explore the challenges and management strategies associated with hip arthroplasty in patients who have experienced a stroke and subsequent failure of fixation treatment for proximal femur fractures. The multi-morbidity of elderly patients and the dependence resulting from a stroke, combined with a proximal femur fracture, present significant challenges for the entire treatment team, including doctors, physiotherapists, nurses, and family members. These challenges are compounded by the patient’s spastic muscle paresis, limited joint mobility, and sensory disturbances. The postoperative complications in stroke patients undergoing hip arthroplasty are exacerbated by factors such as prolonged immobilization, pain, urinary catheter insertion, and postoperative disorientation. These factors significantly reduce the likelihood of regaining independence and increase the risk of further complications, including additional strokes, heart attacks, and even death [13,14]. Timely and safe surgical intervention, followed by early postoperative rehabilitation, are crucial for stroke patients with hip fractures. These measures significantly enhance the chances of regaining independence and improving the overall outcomes [5]. Furthermore, addressing the unique challenges posed by spastic muscle paresis and limited joint mobility in post-stroke patients requires a multidisciplinary approach. This includes comprehensive preoperative planning, effective pain management, and a structured rehabilitation program to mitigate the risks of long-term complications and enhance patient recovery.

## 2. Case Report: Management of a Spastic Limb in Post-Stroke Patient with Hip Fracture

### 2.1. The Patient

This case study describes a 64-year-old man with left-sided hemiparesis and a failed surgical treatment of a pertrochanteric fracture of the proximal femur in the spastic limb. He was admitted to the Department of Rehabilitation and Orthopedics in December 2022 for diagnostic evaluation and treatment planning after five months of non-ambulatory status.

### 2.2. Patient Medical History

The patient’s medical history includes second-degree aortic valve regurgitation, ischemic heart disease, and multiple myocardial infarctions in 2004 and 2007. He also underwent stent implantation in 2004 and suffered a left orbital fracture and left frontal lobe contusion. In 2005, a subdural hemorrhage due to an aneurysm rupture led to left-sided hemiparesis.

### 2.3. Initial Treatment

Following a hemorrhagic stroke in 2005, the patient underwent extensive rehabilitation, focusing on locomotion and upper limb manipulation. Following recovery, he was able to walk independently using a hand cane. His Barthel scale score was 85, indicating slight disability and independence in daily activities, although his Tinetti scale score was 10 out of 28, indicating a high fall risk [15,16]. Table 1 shows the Barthel scale scores, indicating the patient’s functional status before and after treatment. Table 2 displays the Tinetti scale scores, assessing balance and fall risk.

### 2.4. Fracture and Initial Surgery

**Figure 1:**
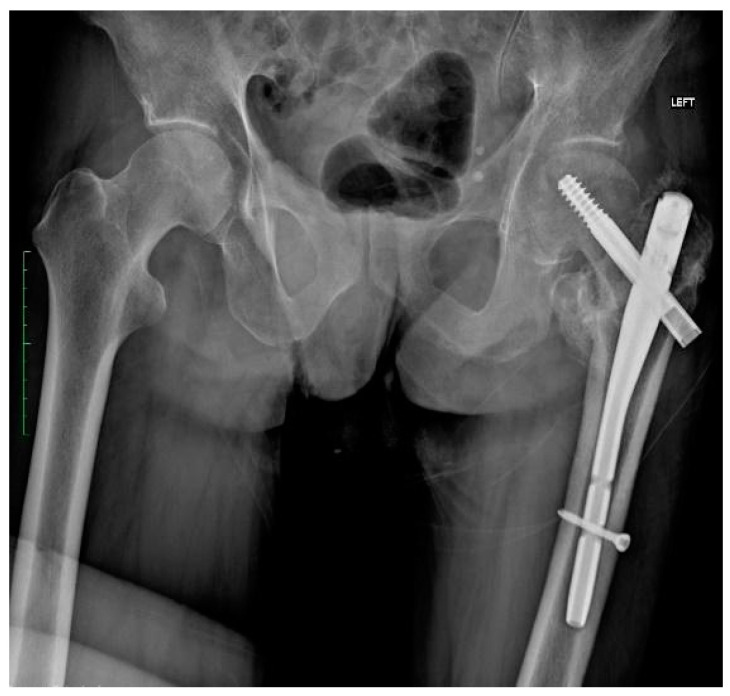
X-ray after surgery. Pertrochanteric fracture of the left lower limb fusion (Gamma nail). Left limb adduction as the result of increased tension of the spastic adductor muscles. Anterior–posterior (AP) view.

In July 2022, the patient came to the emergency room due to severe pain in the left hemiparetic limb and the inability to bear weight on it after a fall from his own height. During the examination, an X-ray revealed a pertrochanteric fracture of the left lower limb. The second day after the fracture, surgery was performed to stabilize the fracture with a Gamma nail. Figure 1 shows the X-ray taken after the initial surgery, illustrating the successful fusion of the fracture. 

Following the initial surgery, the patient experienced a significant decline in mobility, relying entirely on a wheelchair. His Barthel scale score dropped to 40, indicating substantial dependence on others for daily activities. Table 1 and Table 2 illustrate the patient’s functional status and fall risk assessment over time, highlighting the significant decline in independence following the surgery. 

In December 2022, 5 months after the first surgery, the patient consulted a doctor due to pain, a snapping sensation in the area of the operated joint, and a fistula that appeared below the postoperative scar.

### 2.5. Treatment

After admission to the Department of Rehabilitation and Orthopedics, the patient was assessed by a multidisciplinary team of orthopedics, medical rehabilitation, and physiotherapist specialists. The team decided to treat the patient in three stages, preparing him for hip arthroplasty.

**Figure 2:**
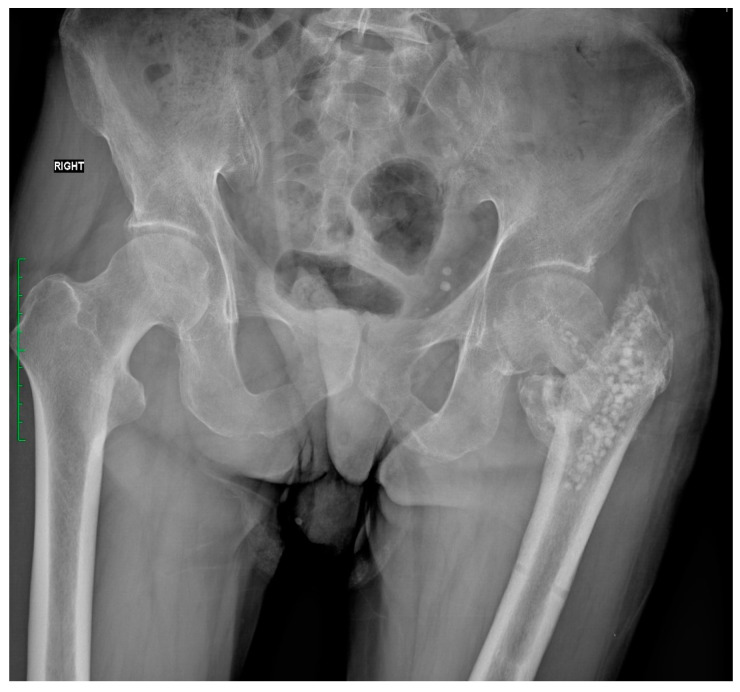
X-ray after surgery. Procedure to remove, fuse, and fill bone defects with absorbable calcium sulfate antibiotic carrier with Gentamicin.

The first stage of treatment, performed in January 2023, involved the removal of the failed fixation and the use of an absorbable calcium sulfate antibiotic carrier with Gentamicin to fill bone defects. This was followed by comprehensive perioperative care, including antibiotic therapy, antithrombotic prophylaxis, and anti-edema management. Figure 2 shows the X-ray taken after the first stage of treatment, illustrating the successful removal of the failed fixation and filling of the bone defects.

Following the surgery, the patient underwent a 6-week intensive rehabilitation program to enhance his general fitness and relearn walking using orthopedic equipment. The patient was discharged from the hospital in good general condition, walking with support for short distances using handrails and a brace limiting the flexion of the left knee joint. Table 3 shows the detailed rehabilitation program for each stage of treatment.

The second stage of treatment, implemented in September 2023, included botulinum toxin (BnT) injections to the spastic muscles and further rehabilitation to improve muscle function and joint mobility.

Before the botulinum treatment, the muscle tension of the paretic limb was assessed as 3 according to the Ashworth scale (mm. gastrocnemius, soleus, posterior tibialis, and adductors). There was also a significant limitation of mobility in the hip joint (flexion–adduction contracture 20 degrees), knee joint (flexion contracture 30 degrees), and a fixed equinus foot position. In the upper limb, the muscle tension of the elbow and wrist flexors was 4 on the Ashworth scale. The left upper limb was assessed as unusable due to severe spasticity and the persistent limitation of mobility of the wrist and finger joints.

The patient’s gait could not be assessed due to pain and fear of falling. After the assessment, the gastrocnemius, tibialis posterior, and adductors were chosen as the injection sites. The BnT treatment (botulinum toxin type A, 1500 IU) was performed with ultrasound guidance to secure an accurate needle position. 

As a result of the drug administration, the spastic muscle tension was reduced to 2 in all the treated muscles. Consequently, the flexion contracture was reduced, and the range of motion in the hip and knee joints was increased, which, combined with the exercises, improved the supporting function of the limb.

**Figure 3:**
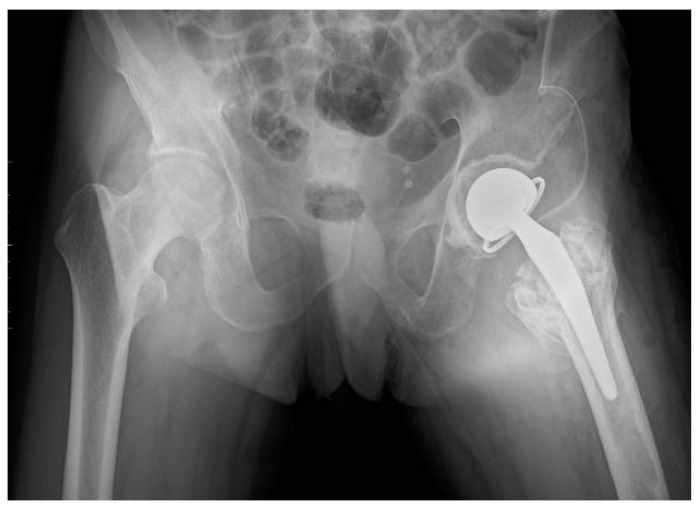
X-ray after surgery. Implantation of a total snap-on endoprosthesis of the left hip joint. AP view.

After finishing a 6-week period of rehabilitation, the patient was qualified for the third and final stage of treatment—the insertion of a complete bipolar snap-on prosthesis with simultaneous tenotomy of the adductor muscles. Figure 3 displays the X-ray after the final surgery, confirming the correct placement of the total snap-on endoprosthesis.

The surgery went without complications, and the patient was able to stand up with the aid of the therapist with a standing walker the first day after the surgery. After two weeks, the initial postoperative rehabilitation treatment was intensified to further improve locomotion. During this time, the patient rehabilitation included Proprioceptive Neuromuscular Facilitation (PNF), passive exercises with a Continuous Passive Motion machine (CPM), and improvement of ambulation with handrails and a hand cane. 

**Figure 4:**
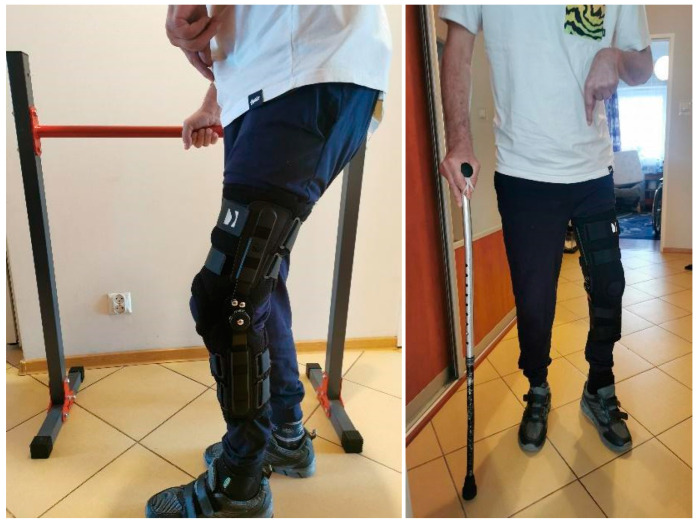
Patient after prosthesoplasty, walking with the help of orthopedic equipment.

**Figure 5:**
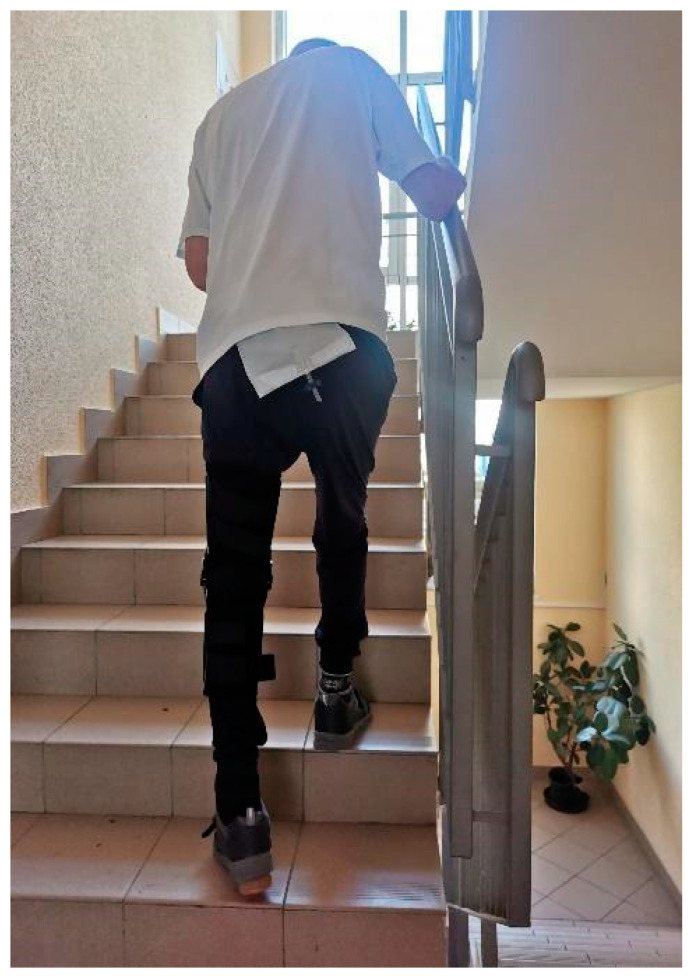
Patient after surgery, walking up the stairs with the help of orthopedic equipment.

**Figure 6:**
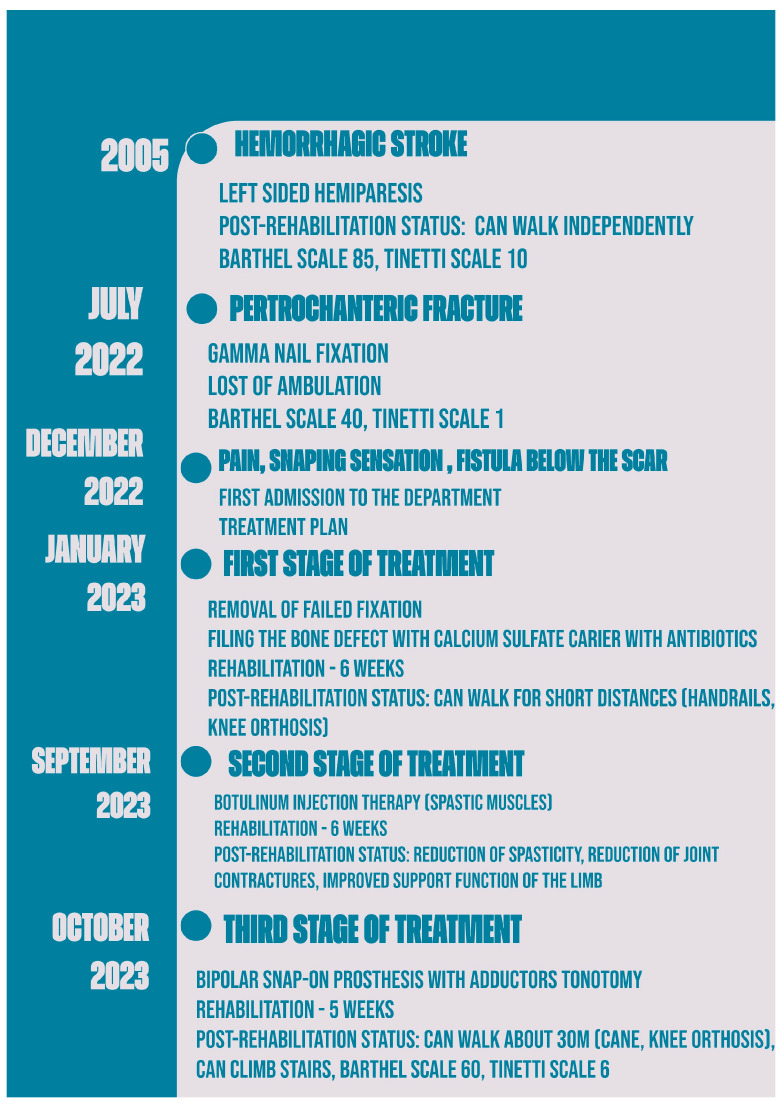
The treatment timeline.

After the final stage of the treatment, which involved the insertion of a complete bipolar snap-on prosthesis and adductor muscle tenotomy, the patient was able to walk short distances (about 30 m) and climb the stairs with support (Figure 4 and Figure 5). His Barthel scale score improved to 60, although his Tinetti scale score remained low at 6, indicating ongoing balance and mobility challenges. The patient was discharged home after five weeks of rehabilitation with recommendations to continue the exercises at home (balance and mobility training to decrease the risk of further falls) and to continue the botulinum therapy every 3 months. Figure 6 displays a detailed timeline of the patient’s treatment.

## 3. Discussion

There is a scarcity of literature addressing the complexities of arthroplasty for proximal femur fractures in patients with spastic hemiparesis. Despite the scarcity of literature, this issue is well-recognized in modern medicine. The absence of standardized surgical protocols for patients with spastic hemiparesis and multiple comorbidities presents a significant medical challenge. The studies indicate that patients with hemiplegia have higher risks of fractures due to factors such as sensory disorders, weakened muscle strength, and spasticity, which impair balance [6,7,8,9,10,11,12,13,14,15,16,17,18,19,20]. Fisher et al. reported that a significant proportion of these patients have vitamin D deficiency and increased bone resorption, predisposing them to osteoporosis and falls [13]. 

Our patient scored 10 on the Tinetti scale following a stroke, indicating a high fall risk and underscoring the need for targeted rehabilitation to improve balance and coordination [21,22,23,24]. The Barthel scale, used to assess patient independence in daily activities, showed a score of 60, indicating moderate disability and partial assistance needs [15].

Pre- and postoperative rehabilitation for hip prosthesis surgery in paralyzed patients is vital for achieving successful treatment outcomes and enhancing their quality of life. The necessity for early and comprehensive physiotherapy following total hip arthroplasty has been demonstrated. The patient evaluations consistently indicate that their quality of life improved following the insertion of an artificial hip and subsequent rehabilitation [25].

Although research has been conducted to establish effective rehabilitation strategies for patients following hip fracture surgery, currently, there are no clear and evidence-based guidelines for managing patients with both stroke and hip fractures [11,26,27]. Developing such standards is essential for improving patient outcomes and reducing complications. Choosing a proper strategy for managing a patient who has experienced both a stroke and a hip fracture presents significant challenges for the whole treatment team. Consequently, our work addresses this issue by proposing pre- and postoperative procedure programs. 

In the existing literature, clear guidelines are lacking for hemiparetic patient hip fracture surgeries, including eligibility criteria, surgery timing, and the approach [28]. The surgical decisions are often influenced by the patient’s functional status, comorbidities, type, and stroke severity [11,29,30].

Studies indicate that patients with hemiplegia stayed in the hospital longer and developed postoperative complications more often than patients without hemiplegia, as well as those related to the use of bone cement and bone substitutes [11,31,32].

Additionally, the choice of surgical approach and the type of artificial hip joint used can impact the success of postoperative rehabilitation. The research indicates that the direct anterior approach in hip surgery, as used in our case, results in fewer postoperative complications and faster recovery compared to the posterolateral approach, although it involves longer operation times and more intraoperative bleeding [18,33,34]. The bipolar snap-on prosthesis, as used in our patient, facilitated early mobilization and reduced the postoperative dislocations, crucial for patients with spastic paresis and limited joint mobility [26]. 

Spasticity is a pathological condition whose main feature is involuntary and prolonged muscle contraction [35,36,37,38]. Several treatments can help to reduce the tone of spastic muscles, including physiotherapy, pharmacotherapy, and the increasingly popular method of botulinum toxin injections. To achieve optimal results, it is essential to combine multiple approaches. Emerging evidence suggests that combining electrical stimulation to the muscles following botulinum toxin injections with gait training is particularly effective [39]. Over the last decade, the intrathecal baclofen (ITB) pump has been increasingly used to treat generalized or regional spasticity refractory to oral medications or injection therapy [40].

To manage spasticity, botulinum toxin injections were administered to the patient’s lower limb muscles, significantly reducing the muscle tone and improving the joint mobility, thereby enhancing the limb’s supportive function.

A prompt and comprehensive management strategy for patients with both stroke and hip fracture should include timely surgical intervention, appropriate prosthesis selection, and an intensive rehabilitation program incorporating botulinum toxin therapy and physiotherapy for optimal recovery and preparing the musculoskeletal system for gait training.

## 4. Conclusions: Challenges and Management Strategies for Hip Fractures in Spastic Paralysis Patients

Treating hip fractures in patients with spastic paralysis is understudied. This complex issue is recognized in modern medicine, yet the absence of standardized protocols for managing surgical treatment in these patients complicates the situation. The research indicates a higher fracture risk in stroke patients due to sensory disorders, reduced muscle strength with spasticity, and balance issues.

The research also indicates that patients with hemiplegia experience longer hospital stays and more frequent postoperative complications, highlighting the need for specialized care protocols. Choosing the appropriate surgical approach, such as the direct anterior approach, and selecting the optimal type of artificial hip joint are critical for successful rehabilitation and reduced postoperative complications. Effective spasticity management requires a multimodal approach, including botulinum toxin injections combined with electrical stimulation and gait training. The use of intrathecal baclofen pumps is also beneficial for patients with refractory spasticity. A staged treatment program, starting with botulinum toxin injections to reduce muscle spasticity, followed by continuous therapeutic rehabilitation, and culminating in hip arthroplasty, significantly enhances the patient outcomes by promoting independent movement and greater autonomy. In conclusion, the management of hip fractures in patients with spastic paralysis requires a multidisciplinary approach and the development of standardized treatment protocols. This case underscores the importance of comprehensive pre- and postoperative rehabilitation to improve patient outcomes. 

Further research is needed to establish standardized rehabilitation protocols for spastic patients undergoing hip arthroplasty. Randomized controlled trials could provide valuable insights into the efficacy of various interventions.

## Figures and Tables

**Table 1:** Barthel scale [15].

Barthel Index Activity	Score in 2006 (after Stroke Year)	Score in 2022 (before Operation)	Score in 2024 (after Operation)
Feeding 0 = unable 5 = needs help cutting, spreading butter, etc., or requires modified diet 10 = independent	10	10	10
Bathing 0 = dependent 5 = independent (or in shower)	5	0	0
Grooming 0 = needs to help with personal care 5 = independent face/hair/teeth/shaving (implements provided)	5	0	0
Dressing 0 = dependent 5 = needs help but can do about half unaided 10 = independent (including buttons, zips, laces, etc.)	5	0	5
Bowels 0 = incontinent (or needs to be given enemas) 5 = occasional accident 10 = continent	10	10	10
Bladder 0 = incontinent, or catheterized and unable to manage alone 5 = occasional accident 10 = continent	10	10	10
Toilet use 0 = dependent 5 = needs some help, but can do something alone 10 = independent (on and off, dressing, wiping)	5	0	5
Transfers (bed to chair and back) 0 = unable, no sitting balance 5 = major help (one or two people, physical), can sit 10 = minor help (verbal or physical) 15 = independent	10	5	5
Mobility (on level surfaces) 0 = immobile or <50 yards 5 = wheelchair-independent, including corners, > 50 yards 10 = walks with help of one person (verbal or physical) > 50 yards 15 = independent (but may use any aid; for example, stick) > 50 yards	15	5	10
Stairs 0 = unable 5 = needs help (verbal, physical, carrying aid) 10 = independent	10	0	5
Total score (0–100)	85/100	40/100	60/100

**Table 2:** Tinetti scale [16].

Tinetti Balance Test	Score in 2006	Score in 2022	Score in 2024
Sitting balance 0 = Leans or slides in chair 1 = Steady, safe	1	1	1
Rises from chair 0 = Unable to without help 1 = Able, uses arms to help 2 = Able, without use of arms	1	0	1
Attempts to rise 0 = Unable to without help 1 = Able, but requires more than 1 attempt 2 = Able to rise in 1 attempt	1	0	1
Immediate standing balance (first 5 s) 0 = Unsteady (staggers, moves feet, trunk sway) 1 = Steady but uses walker or other support 2 = Steady without walker or other support	1	0	1
Standing balance 0 = Unsteady 1 = Steady but wide stance and uses support 2 = Narrow stance without support	1	0	1
Nudged (with patient’s eyes open) 0 = Begins to fall 1 = Staggers, grabs, catches self 2 = Steady	2	0	0
Nudged (with patient’s eyes closed) 0 = Unsteady 1 = Steady	1	0	0
Turning 360 degrees 0 = Discontinuous steps 1 = Continuous steps	0	0	0
0 = Unsteady (grabs, staggers) 1 = Steady	1	0	0
Sitting down 0 = Unsafe (misjudged distance, falls into chair) 1 = Uses arms or not a smooth motion 2 = Safe, smooth motion	1	0	1
Total score (0–16)	10/16	1/16	6/16

**Table 3:** Rehabilitation treatment summary.

**First Stage of Treatment—January 2023** Removal of the failed fixation, filing the bone defects with calcium sulphate antibiotic carrier		
**Phase**	**Rehabilitation Goals**	**Rehabilitation methods**
Phase I Acute postop Days 1–3 after the surgery	- antalgic hip positioning - pain and swelling control - deep vein thrombosis prevention - contractures prevention - early mobility - preparing for independent locomotion	ROM (range of motion) - passive feet exercises - knee and hip joint extension and flexion assisted exercises - CPM In-bed mobility - learning of safe changing the position in bed - learning of safe sitting in bed
Phase II 3–14 days after the surgery	- restoring muscle strength of the operated leg - normalization of muscle tension - transfer training - locomotion training	- Phase I continuation - slight passive stretching of knee and hip joints - knee joint orthosis (2 h 2 times per day, and during night) - transfer from bed to wheelchair training - locomotion with handrails training
Phase III 3–6 weeks after the surgery	- muscle tension normalization - transfer training - locomotion training	- Phases I and II continuation - lower limbs passive-assist pedal trainer with biofeedback - whirlpool lower limbs massage - PNF
**Second Stage of Treatment—September 2023** Botulinum therapy		
**Phase**	**Rehabilitation Goals**	**Rehabilitation methods**
Phase I Day 1	- muscle tension normalization - active verticalization	ROM - hip and knee passive stretching exercises - fascia massage of the calf muscles and adductors - active verticalization with the help of therapist
Phase II 2nd day–6th week after the treatment	- restoring muscle strength - normalization of muscle tension - locomotion	ROM - passive feet exercises - knee and hip joint extension and flexion assisted exercises - CPM - lower limbs passive-assist pedal trainer with biofeedback - knee joint orthosis (2 h two times per day, and during night) - -PNF - locomotion with handrails and with cane training
**Third Stage of Treatment—October 2023** Snap-on Prosthesoplasty with Adductor Tenotomy		
**Phase**	**Rehabilitation Goals**	**Rehabilitation methods**
Phase I Acute postop Days 1–3 after the surgery	- antalgic hip positioning - pain and swelling control - deep vein thrombosis prevention - contractures prevention - early mobility - active verticalization - preparing for independent locomotion - safe changing position education	ROM - passive feet exercises - knee and hip joint extension and flexion assisted exercises - CPM - cold therapy In-bed mobility - learning of safe changing the position in bed - learning of safe sitting in bed - active verticalization with the help of therapist
Phase II Days 3–14	- restoring muscle strength - muscle tension normalization through limb positioning and exercises - locomotion training	Phase I continuation - slight passive stretching of knee and hip joints to prevent contractures - knee joint orthosis (2 h 2 times per day, and during night) - transfer from bed to wheelchair training - locomotion with handrails and with cane training
Phase III 3–5 weeks after the surgery	- restoring muscle strength - muscle tension normalization through limb positioning and exercises - locomotion training	- Phases I and II continuation - lower limbs passive-assist pedal trainer with biofeedback - whirlpool lower limbs massage - PNF - stairs climbing

## Data Availability

The original contributions presented in the study are included in the article; further inquiries can be directed to the corresponding author.
